# Case Report: AL Amyloidosis Severe Restrictive Cardiomyopathy Associated With Multiple Myeloma—Diagnostic Difficulties

**DOI:** 10.3389/fcvm.2022.862409

**Published:** 2022-06-13

**Authors:** Yulia Y. Kirichenko, Irina S. Ilgisonis, Elena S. Nakhodnova, Irina Y. Sokolova, Olga V. Bochkarnikova, Sabina A. Kardanova, Olga V. Lyapidevskaya, Elena V. Privalova, Vladimir I. Ershov, Yurii N. Belenkov

**Affiliations:** ^1^Department of Hospital Therapy No. 1 of the Federal State Autonomous Educational Institution of Higher Education I.M. Sechenov First Moscow State Medical University of the Ministry of Health of the Russian Federation (Sechenov University), Moscow, Russia; ^2^Department of Hematology, University Hospital No. 1 of the Federal State Autonomous Educational Institution of Higher Education I.M. Sechenov First Moscow State Medical University of the Ministry of Health of the Russian Federation (Sechenov University), Moscow, Russia

**Keywords:** multiple myeloma, cardiotoxicity, vasculotoxicity, cardiac amyloidosis, restrictive cardiomyopathy (RCM), speckle tracking ECHO

## Abstract

**Background:**

Cardiac AL amyloidosis as a complication of multiple myeloma (MM) is a formidable life-threatening condition. The first-line therapy for both MM and systemic AL amyloidosis is proteasome inhibitors (PIs). Unfortunately, the use of PIs may lead to cardiovascular toxicity development, which requires specific cardio-oncology supervision.

**Case Report:**

A 57-year-old woman was admitted to a university hospital with clinical manifestation of progressive chronic heart failure. The patient had hypertension and no history of diabetes mellitus, myocardial infarction (MI), stroke, and arrhythmias. After a series of laboratory and instrumental examination methods, MM complicated by cardiac AL amyloidosis was proved. Upon specific cardio-oncology examination (NT-proBNP 4,274 pg/ml), ECHO showed systolic dysfunction, motion abnormalities in LV basal and middle segments, and a typical depositional myocardium pattern (“luminescence”); cardiac MRI revealed restrictive cardiomyopathy and specific hyperenhancement of the ventricles and atria; 24-h ECG showed QS-pattern in leads V1–V3 and unstable ventricular tachycardia (VT) paroxysms. Cardio-oncology consultation showed baseline cardiovascular risk was very high (≥20%), and cardioprotective therapy [iACE/ARBs, beta-blockers (BB), statins] was administered. The patient underwent VCD (bortezomib; cyclophosphamide; dexamethasone) chemotherapy (CMT) program. By the time of publication, the patient had received four CMT courses with a positive oncohematological and cardiovascular effect.

**Conclusion:**

In this clinical case, we described a complication of MM, which was rare according to the severity and manifestation with restrictive cardiomyopathy due to secondary cardiac amyloidosis. The case's features were difficulties in verifying the underlying disease and its own complication, and the complexity of patient management according to modern principles of cardio-oncology.

## Introduction

Multiple myeloma (MM) is a B-cell malignant tumor; morphological substrate—plasma cells producing monoclonal Ig (1). The annual worldwide incidence of MM is growing steadily to 1.5 cases per 100,000 people.

All organs and systems, including the cardiovascular system, are involved in the clinical picture of MM. In addition to the underlying disease, 10–15% of patients with MM may develop formidable complications such as focal or systemic AL amyloidosis (2).

Amyloidosis is a disease caused by extracellular deposition of a specific protein-polysaccharide complex (amyloid) in various organs and tissues, which leads to cell dysfunction, damage, or death (3, 4). According to the United States National Center for Health Statistics, the prevalence of AL amyloidosis is 4.5 cases per 100,000 populations (5). The main target organs in AL amyloidosis are the heart (70–80%), kidneys (74%), liver (27%), and peripheral and autonomic nervous systems (22 and 18%, respectively) (6, 7). Moreover, in only 5% of cases, a rare manifestation of the disease, such as isolated cardiac amyloidosis, is observed (8, 9). AL cardiac amyloidosis may clinically manifest through progressive chronic heart failure (HF): severe rest dyspnea (in 80% of patients), peripheral edema (70%), pleural effusion, or ascites in the later stages (10). Diagnosis of cardiac amyloidosis is complicated and requires a series of laboratory and instrumental examination methods, but the final diagnosis can only be verified morphologically (8, 9).

The primary approach in treating both MM and AL cardiac amyloidosis is inhibition of pathological precursor protein synthesis and plasmocyte proliferation. According to guidelines for the diagnosis and treatment of MM and systemic AL amyloidosis, the first-line therapies are a combination of proteasome inhibitors (PIs; bortezomib, carfilzomib, and ixazomib) with other chemotherapy drugs (cyclophosphamide, melphalan, and dexamethasone) (9, 11).

However, proteasome inhibition occurs not only in pathological plasma cells but also in normal cardiomyocytes and/or endothelial cells, which may result in development of cardiovascular toxicity. Clinical symptoms may manifest through various rhythm/conduction disturbances, ischemia progression including myocardial infarction (MI), and decrease in systolic function (12, 13). Alkylating drugs (cyclophosphamide and melphalan) and glucocorticoids have similar cardiotoxic effects (13). The incidence of HF during bortezomib therapy is relatively low, up to 4%; however, it can increase by up to 15% with simultaneous use of glucocorticoids (13, 14).

The present clinical case describes a patient suffering from MM; diagnostic difficulties were due to manifestation of severe cardiac AL amyloidosis (restrictive cardiomyopathy, biventricular HF, and life-threatening arrhythmias), and the complexity of patient management according to modern principles of cardio-oncology.

## Case Description

A 57-year-old woman was admitted to the hematology department of Sechenov University in January 2021. The patient complained of chest discomfort, shortness of breath during minimal exertion (walking up to 100–200 m), exercise intolerance, rare episodes of heartbeat interruptions without a provoking factor, hypotension (up to 90/55 mm Hg), and weakness.

It is known that the patient suffered from second-grade arterial hypertension (AH) for many years, managed by low doses of iACE, but she had no history of acute MI, stroke, atrial fibrillation/flutter, pulmonary embolism, or HF.

Carpal tunnel syndrome was verified in 2019. In order to exclude hereditary amyloidosis, direct sequencing of the entire coding sequence and regions of exon-intropic junctions of the transthyretin (TTR) gene was performed; pathogenic and probably pathogenic variants of the TTR gene nucleotide sequence were not found. After a neurological consultation, symptomatic therapy with pregabalin was prescribed for several months with a moderate effect.

The patient developed the above complaints in September 2020.

**Figure 1:**
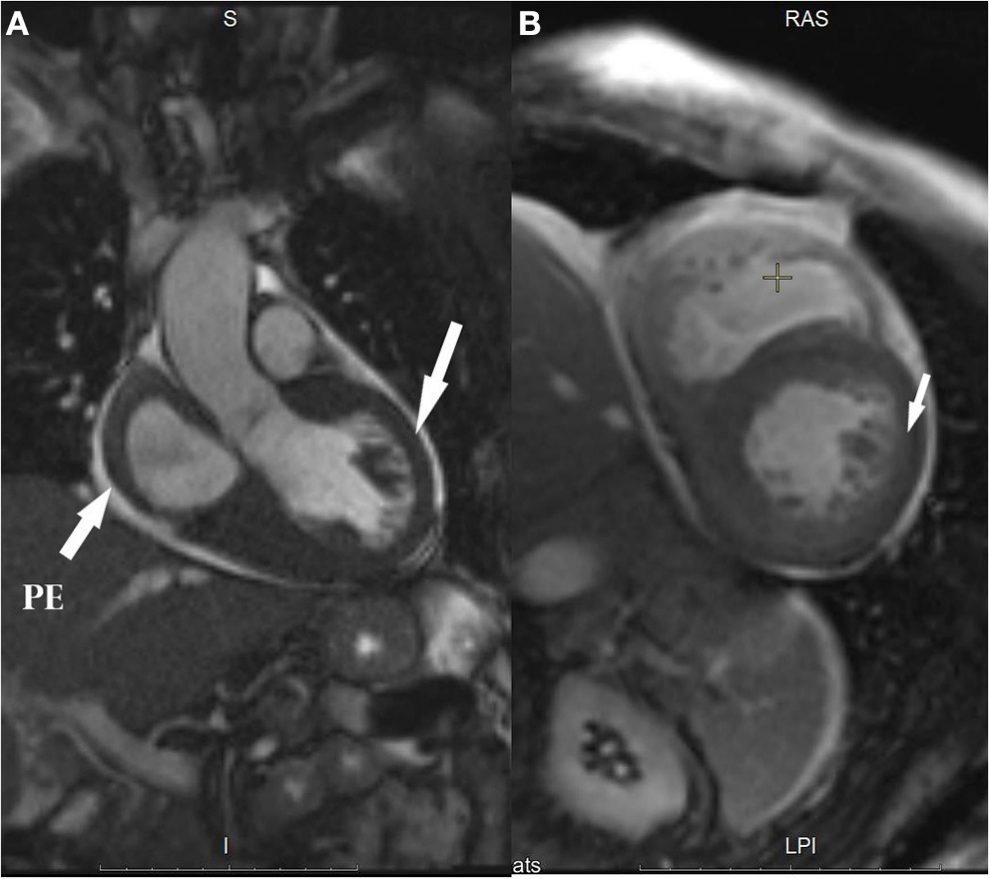
Cardiac MRI images: **(A)** Left ventricle (LV) outflow tract (arrows show ventricular hypertrophy and pericardial effusion, PE); **(B)** Two-chamber views (arrow shows global subendocardial hyperenhancement).

We found the following upon outpatient examination:

- ECG and 24-h ECG showed sinus tachycardia, complete left bundle branch block, QS-pattern in leads V1–V3, frequent supraventricular extrasystoles (SVEs), ventricular extrasystoles (VEs), and unstable VT paroxysms without rhythm pauses or ischemia (at that time rhythm disorders were not interpreted, and anti-arrhythmic drugs were not prescribed);
- ECHO showed left atrium (LA) dilatation, concentric left ventricle (LV) and right ventricle (RV) hypertrophy (no zones of local contractility disorders), decreased global myocardial contractility, ejection fraction (EF) 48%, mitral regurgitation grade 2, tricuspid regurgitation grade 2, moderate pulmonary hypertension, physiological amount of fluid in the pericardial cavity, and increased echogenicity of the LV myocardium;
- We saw a significant increase in blood NT-proBNP level up to 4,274 pg/ml (*N* = 0–125);
- Cardiac MRI with late gadolinium enhancement showed restrictive cardiomyopathy, LV hypertrophy, moderate atria expansion, and specific contrasting of the ventricles and atria myocardium, which did not exclude cardiac amyloidosis (Figure 1).

Outpatient examination continued; serum and urine protein immunochemical study detected κ-type Bens-Jones protein [serum κ- free light chain (κ-FLC) 239 mg/L (*N* = 3.3–19.4), daily proteinuria 1.5 g], secondary hypogammaglobulinemia, increased serum β2-microglobulin levels, and dysproteinemia with α-1/α-2 fraction predominance.

The patient was consulted by a hematologist and in-charged with the above anamnesis. On admission (physical examination) the patient showed no obesity (BMI = 26.9 kg/m^2^), no fever (T 36.7°C), no peripheral lymphadenopathy, no edema, normal lung breathing sounds, no wheezing, Sat O2 98% in room air, muffled heart sounds, arrhythmia due to single EXs, heart rate (HR) 86 bpm, blood pressure (BP) 100/60 mmHg, no hepatosplenomegaly, and no obvious disturbances of organs and systems.

Blood test abnormalities were as follows: AST 37 U/L (*N* = 0–34), γ-GT 123 U/L (*N* = 0–73), CPK 197 U/L (*N* = 0–190); potassium 5.5 mmol/L (*N* = 3.4–5), eGFR (CKD-EPI) 68 ml/min/1.73 m^2^, LDH 590 U/L (*N* = 240–480), cholesterol 5.8 mmol/L (*N* = 3.2–5.6), triglycerides 2.44 mmol/L (*N* = 0.4–1.7), VLDL 95 mmol/L (*N* = 0.19–0.77), HDL.83 mmol/L (*N* ≥ 1.56), troponin T (twice) negative, dysproteinemia with α-1 fraction predominance, secondary hypogammaglobulinemia, M–gradient negative, fibrinogen 5.12 g/L (*N* = 1.8–4), and daily proteinuria 1 g. All other parameters were in normal range.

Whole-body low-dose CT scan revealed no foci of destruction.

Sternal puncture showed an increased amount of plasma cells up to 8%. Thus, according to the bone marrow cytological examination, no convincing data for MM were obtained (11, 15).

Subcutaneous fat and rectal mucosa biopsy with Congo red staining (for the diagnosis of specific amyloid lesions) was negative.

Trepanobiopsy (for final diagnosis verification) showed a morphological picture corresponding to the substrate of plasma cell myeloma; during an additional histochemical study, the amyloid-Congo-red-complex was found.

Thus, according to the European and National guidelines for the diagnosis and management of multiple myeloma and systemic AL amyloidosis, these diseases were confirmed (11, 15).

**Figure 2:**
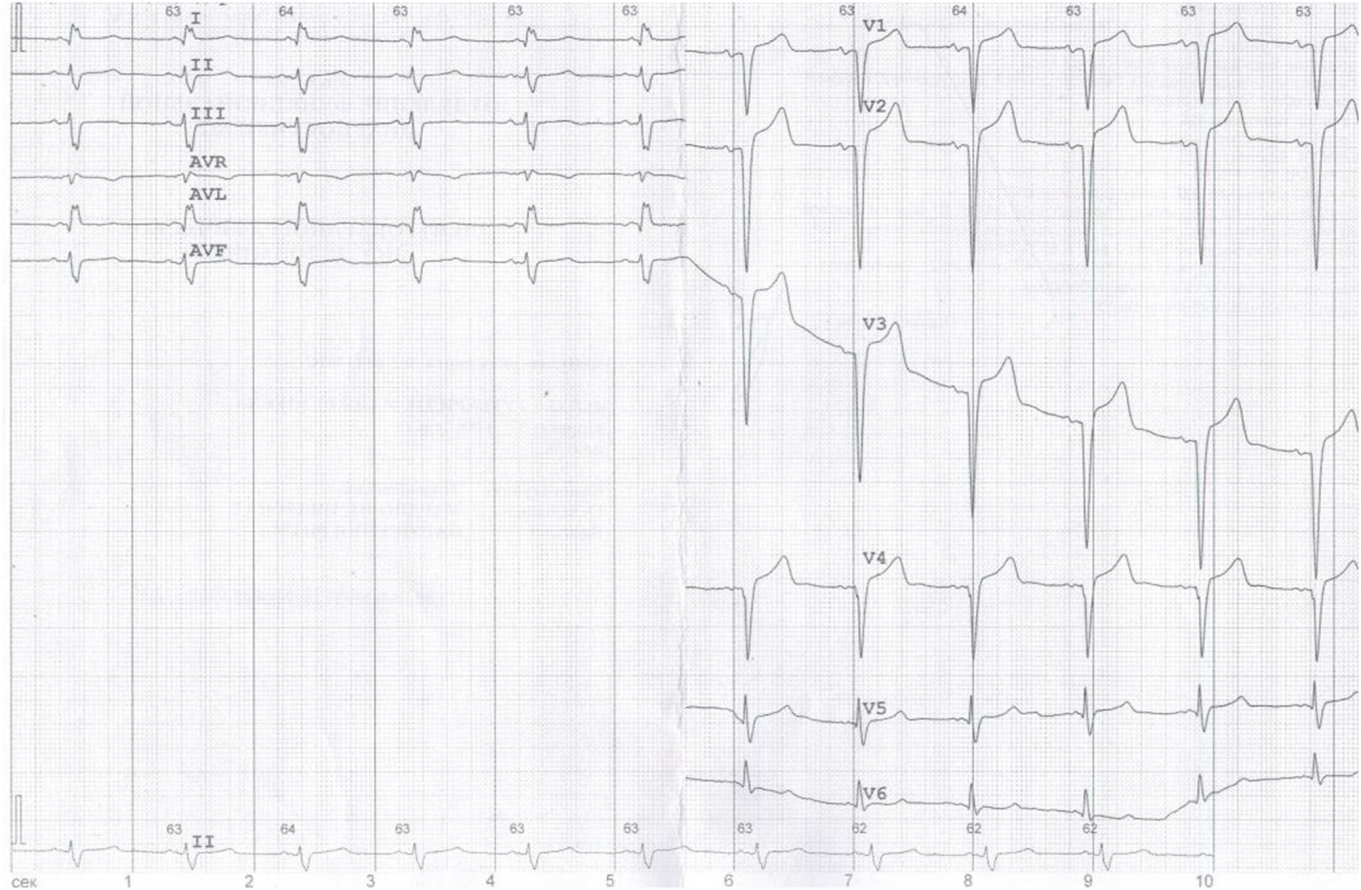
ECG: Sinus tachycardia, heart rate (HR) 63 bpm, left anterior fascicular block, QS-pattern in leads V1–V3.

**Figure 3:**
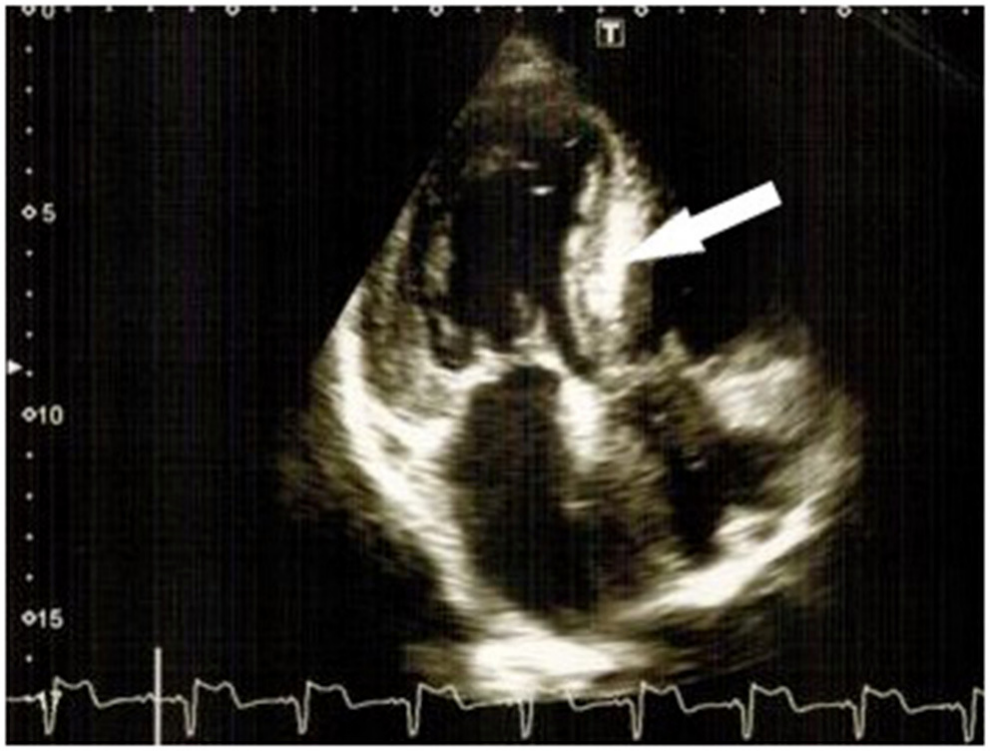
“Characteristic luminescence” of the interventricular septum (bold arrows) and left ventricle hypertrophy.

**Figure 4:**
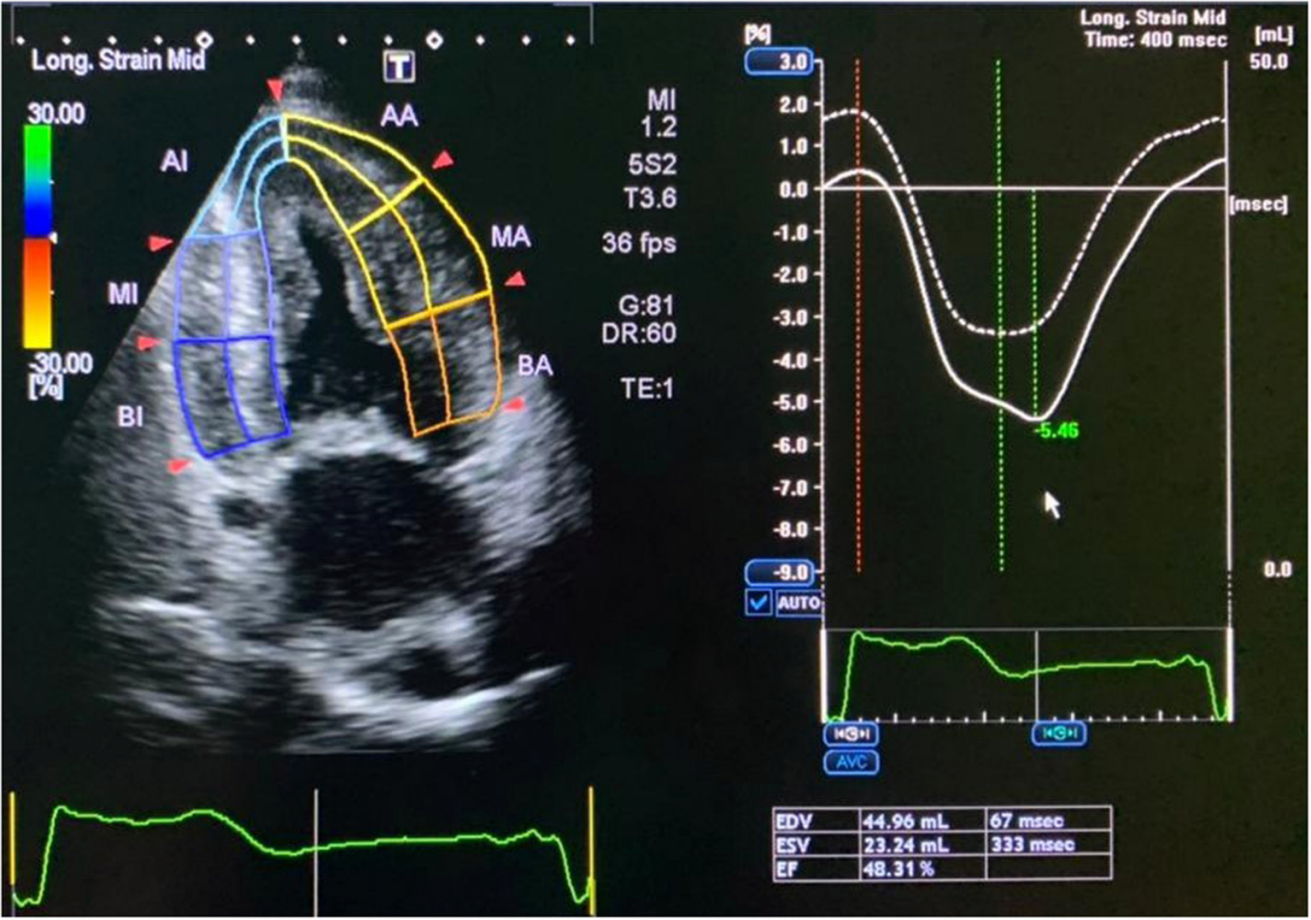
Speckle tracking for two-chamber view.

Additionally, due to the patient's cardiac complaints and signs of heart involvement, before starting potentially cardiotoxic cancer therapy, the patient was further examined (ECG, 24-h ECG, and 2D speckle tracking ECHO) (Figures 2–4) and consulted by a cardio-oncologist.

A 24-h ECG showed a sinus rhythm with average day HR 78 bpm, average night HR 77 bpm, SVEs—total 420, max per hour−39, six couplets, four paroxysms of SVT up to 2 s, VEs—total 227, max per hour−89, 15 couplets, paroxysms of unstable VT (3 paroxysms: 1 triplet, other consists of 4–5 beats), no inducible myocardial ischemia, no rhythm pauses longer than 2 s. 2D Speckle tracking ECHO: significant concentric LVH (average 17 mm, *N* = 10 mm) and RVH (7 mm, *N* = 5 mm), decreased LV systolic function [biplane EF = 48–50% (Simpson), dp/dt = 1,185 mmHg, GLS = −5% (*N* > 18%)] and RV [TAPSE = 1 cm (*N* > 1.7)], motion abnormalities in LV basal and middle segments, restrictive type of LV diastolic dysfunction (E/A = 2.9), LA dilatation (4.2 cm, volume 75 ml, *N* < 55 ml), mitral/tricuspid valve leaflet thickening, moderate regurgitation, mild pulmonary hypertension (estimated sPAP = 37–40 mmHg, *N* < 30 mmHg), mild pericardial effusion (1–2 mm), typical depositional myocardium pattern (“luminescence”), and characteristic cardiac amyloidosis (Figure 4).

Consultation with a cardio-oncologist (ESC HFA/ICOS 2020) showed a baseline cardiovascular risk for development of CMT cardiovascular toxicity was very high (≥20%): previous cardiovascular disease (CVD; HF, cardiac amyloidosis, baseline LVEF < 50%, arrhythmia, LVH); elevated baseline NT-proBNP; CV risk factors (AH, dyslipidemia, high-dose dexamethasone) (16, 17). According to modern cardio-oncology guidelines, cardioprotective drugs should be administered to very high-risk patients (iACE/ARBs, BB, statins) (13, 17). In the case of congestive HF, treatment was carried out according to the ESC Guidelines for the diagnosis and treatment of heart failure 2016 (18).

Thus, based on the results of laboratory and instrumental examination, the diagnosis was verified: multiple myeloma, diffuse form, with the secretion of κ-type light chains; Bence-Jones κ-type proteinuria, stage III (ISS), Durie-Salmon stage I; secondary AL amyloidosis; restrictive cardiomyopathy; cardiac rhythm and conduction disorders—SVTs, VEs, paroxysms of unstable VT, and left anterior fascicular block; heart failure with mid-range EF, functional class III (NYHA); arterial hypertension stage II, degree 2, high CV-complication risk; dyslipidemia (treated by statins); atherosclerosis of the aorta, aortic, mitral, and tricuspid valves, CKD C2 (KDIGO). According to the ESC Position Paper on Diagnostic and Treatment of Cardiac Amyloidosis 2021, there was no doubt that cardiac amyloidosis was the main reason for the CV manifestation in this patient: LV wall thickness was ≥12mm + 1 “red flag” (hypotension, if previously hypertensive, proteinuria, carpal tunnel syndrome, subendocardial/transmural late gadolinium enhancement, reduced GLS, pseudo Q-waves on ECG) and the extracardiac biopsy was positive for amyloids. In this case, there were no indications for cardiac biopsy (6).

Following the guidelines for the treatment of MM complicated by AL amyloidosis (15) and considering exceptionally high cardiotoxicity risk, the patient was scheduled for the VCD program [VELCADE (bortezomib), cyclophosphamide, dexamethasone]. In addition, recommended cardioprotective medications were prescribed: anti-arrhythmic: sotalol 60 mg daily, MRA: spironolactone 50 mg daily, diuretic: torasemide 10 mg daily, metabolic: trimetazidine 80 mg daily, hypolipidemic: atorvastatin 10 mg daily, and anticoagulant: apixaban 5 mg twice a day (according to hematological indications when using high doses of dexamethasone). Due to the high risk of hypotension, iACE/ARB administration was withdrawn until optimal BP levels were established. Class III antiarrhythmic drugs were chosen in order to prevent life-threatening arrhythmias (paroxysmal VT). There was an attempt to administer amiodarone, but it was not tolerated by the patient (extreme systemic hypotension, nausea, vomiting, and dizziness). Thus, sotalol was the only option in this case with regular ECG control (throughout the whole follow-up period no QTc prolongation was registered). There were no absolute indications for implanting a cardioverter-defibralator. The above management approach concerning specific anticancer and cardiac therapy allowed the patient to successfully receive the first CMT course without complications and/or intercurrent infections. In the control blood tests, cytopenia was not noted, and daily proteinuria was absent. The patient was discharged.

By June 2021, the patient had received four VCD chemotherapy courses at the same dosage. The second cycle was interrupted because of SARS-CoV infection, complicated by bilateral polysegmental viral pneumonia (CT-stage 2) with mild respiratory insufficiency and unilateral pleural infusion. Despite the underlying disease and because of COVID-19, the patient received tocilizumab 400 mg and glucocorticoids with a positive effect. Unfortunately, concerning CVD status, COVID-19 resulted in biventricular HF exacerbation, worsening of dyspnea and weakness, peripheral edema, exercise intolerance, NT-proBNP of 8,699. pg/ml, and LV EF = 49%. Correction of cardioprotective therapy was performed by a cardio-oncologist (transition to temporary intravenous diuretic therapy, increasing doses of spironolactone and sotalol) with a positive effect. There was still no opportunity to start iACE/ARBs due to the high hypotensive risk.

Control examination after four CMT courses showed a positive effect: no dysproteinemia with α-1/α-2 predominance, γ-globulin level within the normal range, CRP 6.9 mg/L (as post-COVID-19), NT-proBNP decreased to 4,623 pg/ml, M-gradient undetectable by blood immunoelectrophoresis, Bence-Jones daily proteinuria of only. One gram, 24-h ECG: sinus rhythm with average HR 82 bpm, SVEs: total 380, 13 couplets, 5 paroxysms of asymptomatic unstable SVT, VEs: total 725, 0 couplets, one episode of unstable VT (triplet), Speckle tracking ECHO:GLS increased up to −11%, and EF = 51%.

Such a multidisciplinary approach to patient management, active monitoring of the cardiovascular system's state, and in-time therapy correction made it possible to continue the effective and recommended CMT without delay/withholding.

In the future, re-inpatient examinations are planned for subsequent CMT courses, follow-up for MM, AL amyloidosis, and cardiac control.

## Discussion

This clinical case demonstrates the difficulties in verifying MM due to the lack of proven criteria for its diagnosis and the manifestation of the disease predominantly with cardiac complaints. The severity of the patient's condition is due to complications of MM, such as cardiac AL amyloidosis (restrictive cardiomyopathy, biventricular congestive HF, and life-threatening rhythm disorders). The presence of cardiac amyloidosis indicates a worse prognosis compared with amyloidosis damage to other organs. Predictors of an unfavorable outcome are congestive HF, arrhythmia, renal failure, and involvement of two or more visceral organs in the pathological process. The median survival rate in patients with cardiac AL amyloidosis and HF does not exceed 67 months (8, 9). In addition, using CMT with known cardiovascular toxic effects may further worsen the prognosis in these patients. On the other hand, CMT is currently the only treatment option for MM complicated by AL amyloidosis. Reducing the risk of such therapy becomes possible only with a multidisciplinary approach to the management of these patients by oncologist/hematologist and cardiologist/cardio-oncologist.

Thus, high-quality examination (morphological, immunological, and immunohistochemical) makes it possible to verify the diagnosis and give in-time specific treatment. Moreover, patients with cancer and known CVD and/or cardiovascular risk factors are recommended to be assessed for baseline cardiovascular risk before initiating potentially cardiotoxic cancer therapies. All patients in high/very high-risk groups are needed to be consulted by a cardiologist/cardio-oncologist, and cardiological assessment should include ECG, speckle tracking ECHO, and cardiac biomarkers (hsTr, BNP/NT-proBNP) (17, 19, 20). In the case of a high/very high-risk patient, it is recommended to start cardioprotective drugs: iACE/ARBs, BB, or statins (13, 17). In recent publications, novel cardioprotective strategies were proposed based on SGLT-2 inhibition and interleukin-1 blockers (21, 22). However, for now, these are promising directions that need further investigation and a solid evidence base. Only this approach will help improve the quality of life and survival rate of these complex and prognostically unfavorable patients.

## Conclusion

In this clinical case, we described a rare complication of multiple myeloma and severe restrictive cardiomyopathy due to secondary cardiac amyloidosis. The case's features were difficulties in verifying the underlying disease and its complications and the complexity of patient management according to modern principles of cardio-oncology.

## Data Availability Statement

The original contributions presented in the study are included in the article/supplementary material, further inquiries can be directed to the corresponding author/s.

## Ethics Statement

The studies involving human participants were reviewed and approved by the Local Ethics Committee of the Federal State Autonomous Educational Institution of Higher Education I.M. Sechenov First Moscow State Medical University of the Ministry of Health of the Russian Federation (Sechenov University). The patients/participants provided their written informed consent to participate in this study. Written informed consent was obtained from the individual(s) for the publication of any potentially identifiable images or data included in this article.

## Author Contributions

All authors listed have made a substantial, direct, and intellectual contribution to the work and approved it for publication.

## Conflict of Interest

The authors declare that the research was conducted in the absence of any commercial or financial relationships that could be construed as a potential conflict of interest.

## Publisher's Note

All claims expressed in this article are solely those of the authors and do not necessarily represent those of their affiliated organizations, or those of the publisher, the editors and the reviewers. Any product that may be evaluated in this article, or claim that may be made by its manufacturer, is not guaranteed or endorsed by the publisher.

## Abbreviations

AH: arterial hypertension
ARBs: angiotensin II receptor blockers
BB: beta-blockers
BMI: body mass index
BNP/NT-proBNP: brain natriuretic peptide/N-terminal-pro brain natriuretic peptide
BP: blood pressure
CMT: chemotherapy
CVD: cardiovascular disease
ECG: electrocardiogram
ECHO: transthoracic echocardiography
EF: ejection fraction
GLS: global longitudinal strain
HF: heart failure
HR: heart rate
hsTr: high sensitive troponin
iACE: angiotensin-converting-enzyme inhibitor
Ig: immunoglobulin
LA: left atrium
LV: left ventricle
LVH: left ventricle hypertrophy
MI: myocardial infarction
MM: multiple myeloma
MRI: magnetic resonance imaging
PIs: proteasome inhibitors
RV: right ventricle
RVH: right ventricle hypertrophy
SVE: supraventricular ectopic beat
VE: ventricular ectopic beat
VT: ventricular tachycardia.
